# Synthesis, application and molecular docking of modified cellulose with diaminoguanidine as complexing agent for selective separation of Cu (II), Cd (II) and Hg (II) ions from alum sample

**DOI:** 10.1038/s41598-024-67218-z

**Published:** 2024-07-22

**Authors:** Heba E. Saad, Yusif S. El-Sayed, Gaber M. Abu El-Reash, Mohamed gaber, Mohamed A. Hashem

**Affiliations:** 1 Department of Chemistry, Faculty of Science, Tanta University, Tanta, 31527 Egypt; 2 Department of Chemistry, Faculty of Science, Mansoura University, Mansoura, 35516 Egypt

**Keywords:** Silylating agent, Metal ions, Silanized cellulose, Solid phase extraction, Diaminoguanidine modified cellulose and molecular docking, Chemistry, Analytical chemistry

## Abstract

A new modified cellulose with diaminoguanidine (Cel-Gua) synthesized for specific recovery of Cu (II), Cd (II), and Hg (II) from the alum sample. Cellulose was silanized by 3-chloropropyltrimethoxysilane and then was modified with diaminoguanidine to obtain N-donor chelating fibers. Fourier transform-infrared spectroscopy, scanning electron microscopy, X-ray diffraction, zeta potential, electrons disperse X-ray analysis, elemental analyses (C, H and N), and thermogravimetric analysis were used for characterization. Factors influencing the adsorption were thoroughly examined. Under the optimal conditions, the Cel-Gua sorbent displayed maximum adsorption capacities of 94.33, 112.10 and 95.78 mg/g for Cu (II), Cd (II), and Hg (II), respectively. The sorption process of metal ions is equipped by kinetic model PSO and Langmuir adsorption isotherm. The calculated thermodynamic variables confirmed that the adsorption of Cu (II), Cd (II) and Hg (II) by Cel-Gua sorbent is a spontaneous and exothermic process. In our study, we used the molecular operating environment software to conduct molecular docking simulations on the Cel-Gua compound. The results of the docking simulations showed that the Cel-Gua compound displayed greater potency and a stronger affinity for the Avr2 effector protein derived from Fusarium oxysporum, a fungal plant pathogen (code 5OD4). The adsorbent was stable for 7 cycles, thus allowing its safe reutilization.

## Introduction

Cellulose, being the most prevalent polymer in the natural world, could be sourced from various origins, including bacteria and plants. The vast bounty of chlorophyll-containing plants necessitates the utilization of chemical processes for the extraction and whitening of cellulose when producing paper and its derivatives on a large scale. On a more limited scale, as well cellulose can be derived into thin films^1–3^ and extracted from specific bacteria^4–8^, recent studies have focused on investigating cellulose for the production of biofuel ethanol^9–12^.

Due to the cellulose being the most and largest abundant biopolymer, additionally it has been suggested as a potential material for applications such as solid-phase extraction processes targeting for eliminating toxic metal ions in aqueous samples^4,13–15^. The cellulose's capacity to perform this function is derived from the existence of hydroxyl groups in its structure, which possess the capability to participate in silylation and/or chlorination reactions^16^. These reactions lead to the formation of the reacting structure containing chlorine atoms appropriated throughout the polymer chain^17^. Silylated and/or chlorinated cellulose exhibits reactivity towards a wide array of molecules containing phosphorus, nitrogen, carboxylic acids, sulfur and other functional groups^18,19^.

During the modification processes, molecules that include essential centers (atoms via pairs of unbound electrons) and groups that are established in the cellulose structure may play the role of ion exchangers. As a result, the cellulose that is produced can be employed for solid/phase extraction of metal ions^20–22^.

This sort of method facilitates the separation of the analytes (metal ions) from the structure of the matrix and guarantees more accurate measurement. Currently, Pre-concentration methods employ a wide range of chemically modified organic materials^15,23,24^ and other inorganic^25^. In many circumstances, these methods lower cost of the analysis, which may be performed using economical equipment^26–28^.

A lot of complexed agents are employed in precipitation and/or co-precipitation processes for separating and removing metal types in aqueous and non-aqueous samples^29–32^. Nevertheless, since the complexing agent and the analyte are in the exact same state as the structure of the matrix, separation may be a convoluted and lengthy phase^22,33,34^.

The chelating agent aminoguanidine is frequently utilized in the spectroscopic analysis of metal ions in aqueous solutions, including but not limited to mercury, copper, cadmium, nickel, cobalt, and iron^35–37^.

The N-aminoguanidine is commercial available and very interesting ligand so it can be used for the modification of cellulose for producing new chelating fibers Cel-Gua which has high selectivity and higher adsorption capacity comparing with different modified cellulose as shown in Table 6. The future perspectives in the present study are the using different aminoguanidine moieties (aminoguanidine, diaminoguanidine and triaminoguanidine) and comparing between adsorption capacities showing the rule of increasing the aminoguanidine moieties. Subsequently, the resultant material was utilized to the solid/phase extraction of copper, cadmium, and mercury in aqueous samples. Characterization of the generated material was conducted through various methods, including infrared spectroscopy, thermal analysis, zeta potential, X-ray diffraction, electrons disperse x-ray (EDS) analysis, elemental analyses (C, H and N), and scanning electron microscopy, with a focus on variables associated with the adsorption process. The application of the material involved metal ions pre-concentration in an alum sample, followed by analysis using atomic absorption spectroscopy to obtain results.

## Materials and methods

### Reagents and solutions

Metallic solutions were all formulated utilizing the corresponding high-grade salts from (Merck, Germany), which were then dissolved in ultra-pure water. The standard solutions employed in atomic absorption spectroscopy were made through the dilution of stock solutions with a concentration of 1000 mg/L. Dilution of the concentrated solutions facilitated the preparation of solutions containing HNO_3_, HCl, and H_3_C_2_OOH acids. Dilute Sodium Hydroxide (NaOH) solutions were formulated through the process of diluting the concentrate base sourced from (Merck, Germany), using ultra-pure water. The complexing agent, diaminoguanidine monohydrochloride (CH_7_N_5_.HCl) (Sigma–Aldrich), was utilised without previous treatment or elimination. 3-chloropropyl trimethoxysilane (C_6_H_15_ClO_3_Si) (Sigma- Aldrich). 4-(2-pyridylazo) resorcinol monosodium salt monohydrate (Alfa Aesar). All solvents, such ethanol or dimethylformamide (Sigma- Aldrich). Commercial cellulose was utilized without previous treatment (Sigma Cellulose Type 50, USA) even for drying.

### Preparation of Cel-Gua (modified cellulose)

**Figure 1:**
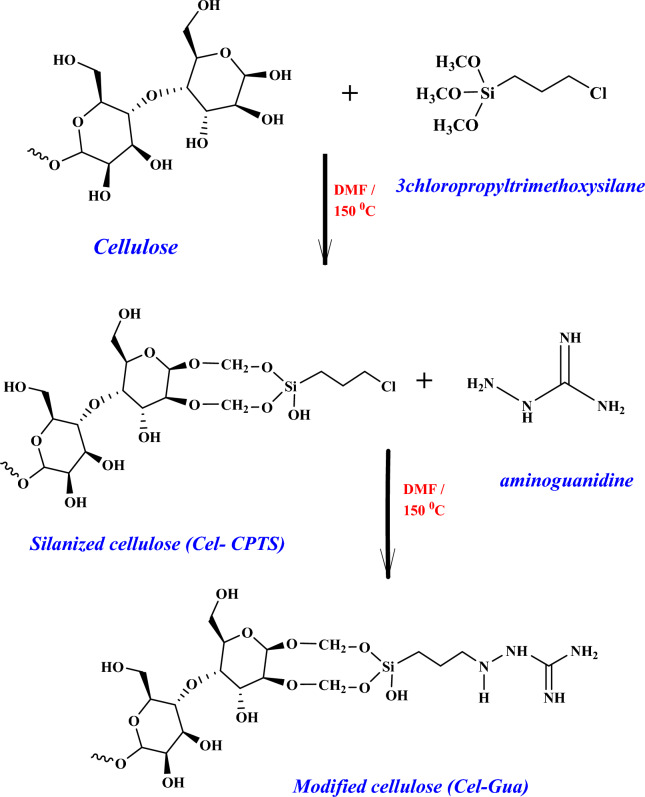
Diagram of the commercial cellulose modification reaction with the diaminoguanidine molecule.

Primarily, 2.0 g of commercial cellulose were exposed to vacuum oven treatment at 100 °C for duration of 24 h to eliminate any potentially adsorbed water molecules. Subsequently, a suspension of 2.0 g of cellulose in (30 mL) of DMF was prepared within a reaction flask, followed by the addition of 4 mL of (3-chloropropyltrimethoxysilane). The ensuing reaction took place under a nitrogen atmosphere, with reflux occurring at 150 °C in a glycerin bath for a period of 48 h. The resultant product underwent washing with DMF, followed by placement in ethanol for 24 h within Soxhlet extraction system and subsequent drying in oven at 150 °C. In subsequent phase of the reaction, 2 g of diaminoguanidine were dissolved within 20 ml DMF, with the addition of cellulose containing the silylating agent (Cel-CPTS). Following that, the mixture was heated and stirred at 150 °C for 48 h. Upon completion of this duration, the end product (cellulose) was modified by diaminoguanidine (Cel-Gua), underwent washing with DMF, was placed in a soxhlet extraction system with ethanol for 24 h and was then dried in the oven. The end product was stored in a desiccator up till further use. A schematic representation of the series of reaction phases is displayed in Fig. 1.

### Equipment and instruments

Throughout the adsorption studies, the samples were shaken in a 50 mL bottle. The metal ions were measured using an atomic absorption spectrometer, (Shimadzu AA6800) fitted with a graphite-furnace or flame portion, as required. The monochromator of the instrument was modified to 510, 510, and 522 nm, which are extremely susceptible resonance patterns of copper, cadmium, and mercury, respectively. The Fourier transform infrared (FTIR) spectrum was documented with an IR spectrophotometer, (Perkin Elmer Spectrum One, USA) at an elimination of 4 cm^−1^, through acquiring the KBr standard disk method. The XRD pattern was acquired via a PANalytical Empyrean Rigaku DMAX-2200 diffractometer, with mono-chromatic Cu Kα1 radiation (λ = 1.540598 Å and 2θ: among 4° and 90°). TGA was accomplished utilizing Shimadzu DTG-60H equipment in an N_2_ environment and at a rate of heating of 10 °C min^−1^. The morphology and structure of the (Cel-Gua) sample, and Energy-dispersive X-ray spectroscopy (EDX) before and after adsorption metal ions were examined with field emission scanning electron microscopy (FESEM) equipment (ZEISS Supra 35VP, Germany) at an acceleration voltage of 5.0 kV. The zeta potential of the (Cel-Gua) solution was determined utilizing a Malvern Zetasizer Nano-ZS particle analyzer at different pH values. Elemental analysis of Cel, Cel-CPTS and Cel-Gua was performed using a Costech (ECS-4010) elemental analyzer.

### Adsorption test

For that reason, the metal ion stock solutions with a concentration of 1000 mg/L were generated by dissolving 0.982 g of copper sulfate pentahydrate (CuSO_4_.5H_2_O), 0.179 g of cadmium chloride monohydrate (CdCl_2_. H_2_O), and 0.135 g of mercuric chloride (HgCl_2_), separately in distilled water. For the batch experiment, all of the required solutions were prepared by diluting the stock solutions with deionized water. Following that, the adsorption experiments were performed to explore one or more differences in metal ion adsorption on the adsorbent Cel-Gua across the optimal conditions of pH (3–9), concentration of metal ions (25–200 mg/L), biosorbent dose (0.02–0.15) g and the time of the adsorption process (30–300 min) at ambient temperature. The original pH of the solution was modified from pH_2_ to pH_9_ for the evaluation of altering pH of the solution via adding droplets of 0.1 M NaOH or 0.1 M HCl. The natural water samples were generated in a volume of 50 mL, and portions of each sample were pre-concentrated under optimum and established conditions. The adsorption investigation was carried out by removing portions of the samples at various times and measuring the remnant concentration with a UV–visible spectrophotometer, and the capacity for adsorption (q_e_) and the removal efficiency of the biosorbent at the state of equilibrium were calculated utilizing Eqs. (1) and (2), accordingly:

1 $$ {\text{q}}_{{\text{e}}} = \frac{{\left( {{\text{C}}_{{\text{i}}} - {\text{C}}_{{\text{e}}} } \right){\text{V}}}}{{\text{m}}} $$

2 $$ {\text{Adsorption}}\;\left( {\% \;{\text{removal}}\;{\text{efficienccy}}} \right) = \frac{{\left( {C_{i} - C_{e} } \right)}}{{C_{i} }} \times 100\% $$

In this context, q_e_ symbolizes the adsorption capacity (mg/g), while C_i_ and C_e_, individually, denote the concentrations at the initial and equilibrium phases (mg/L) of the adsorbate. On the other hand, V and m are used to represent the solution volume (L) and adsorbent mass (g), respectively.

**Figure 2:**
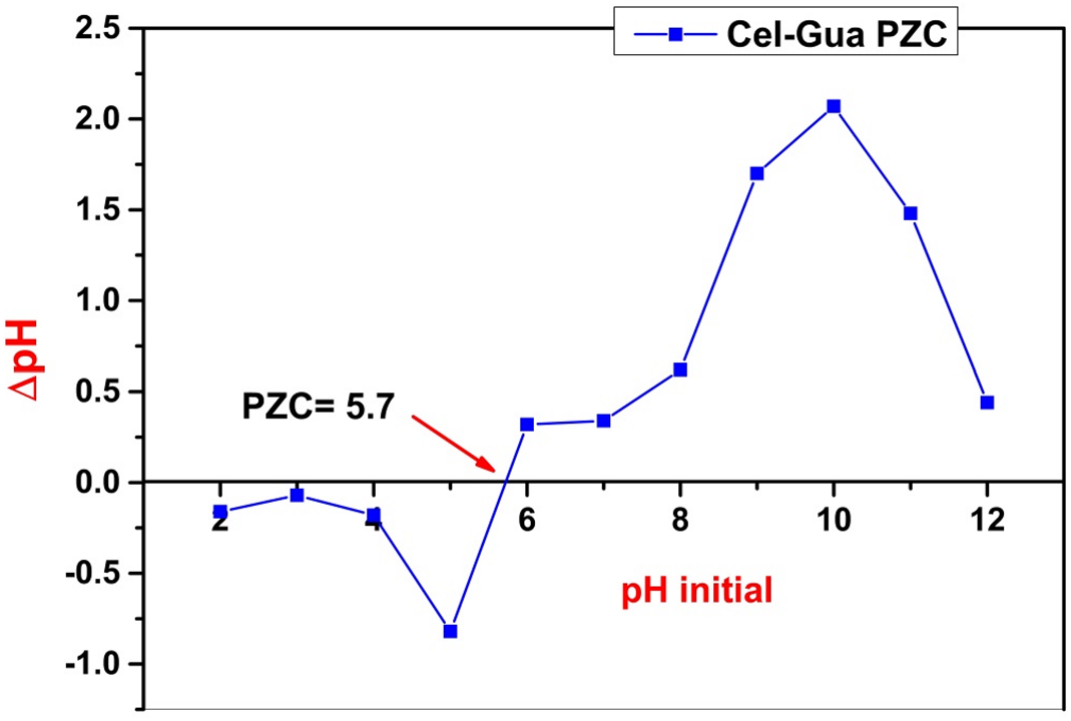
Point of zero charge of Cel-Gua.

The point of zero charge (pH_PZC_) of Cel-Gua was determined as follows: 0.05 g of the Cel-Gua adsorbent was added to a 50 ml of pH adjusted by KCl (0.01 M) solution that varied from 2 to 12 and the mixtures were allowed to shake at the equilibrated shaker for 24 h. 0.1 M of HCl and 0.1 M of NaOH were utilized for KCl pH adjustment. After the shaking, the final pH was recorded and ΔpH was measured as in the following equation (ΔpH = pH_i_ − pH_f_) and was plotted against the initial pH (pH_i_). The pH_pzc_ value is the cross point where the curve ΔpH versus pH_i_ crosses the line ΔpH = 0^38^. Point of zero charge (pH_pzc_) is generally described as the pH at which the net charge of the adsorbent surface is equal to zero in an aqueous solution during the adsorption of ionic species. In this study, a plot for pH_pzc_ determination of Cel-Gua is shown in Fig. 2, which demonstrates the variation of the ΔpH value (pH_initial__pH_final_) of Cel-Gua as a function of the pH_initial_. The pH_pzc_ of Cel-Gua is found to be 5.7. This illustrates that at pH less than 5.7, the Cel-Gua surface is considered to have positive charges.

### Adsorption isotherms

Adsorption isotherm modeling was investigated to establish the quality of the interaction between the adsorbent Cel-Gua compound and the adsorbate metal ions. It is either a chemical or physical interaction. Both Langmuir and Freundlich were used to describe the equilibrium adsorption properties. Equation (3) illustrates Langmuir’s isotherm.

3 $$ {\text{C}}_{{\text{e}}} /{\text{q}}_{{\text{e}}} = \left( {\left( {{1}/{\text{K}}_{{\text{l}}} {\text{q}}_{{{\text{max}}}} } \right) + \left( {{\text{C}}_{{\text{e}}} {\text{q}}_{{{\text{max}}}} } \right)} \right) $$

In this study, q_max_ is used to denote the maximum capacity of the adsorption (mg/g), while K_L_ (L/mg) represents the constant of Langmuir’s isotherm, indicating the affinity among metal ions and test beads. The parameter R_L_, which is dimensionless, serves as the Langmuir isotherm constant to assess the adsorption feasibility, whether it is favorable (0 < R_L_ < 1), irreversible (R_L_ = 0), linear (R_L_ = 1), or unfavorable (R_L_ > 1).

The linear form of Freundlich’s isotherm is shown in Eq. (4).

4 $$ {\text{logq}}_{{\text{e}}} = {\text{logK}}_{{\text{F}}} + {1}/{\text{n}}\;\left( {{\text{logC}}_{{\text{e}}} } \right) $$

Since q_e_ signifies the metal ion equilibrium adsorption capacity, whereas C_e_ signifies the metal ion equilibrium concentration (mg/L), the constant K_F_ (L/mg) is utilized in assessing the adsorption capacity, known as Freundlich’s constant. Additionally, 1/n denotes the adsorption strength, with the value of 1/n indicating whether the adsorption process is unfavorable (1/n > 2) or favorable (0.1 < 1/n < 0.5).

**Figure 3:**
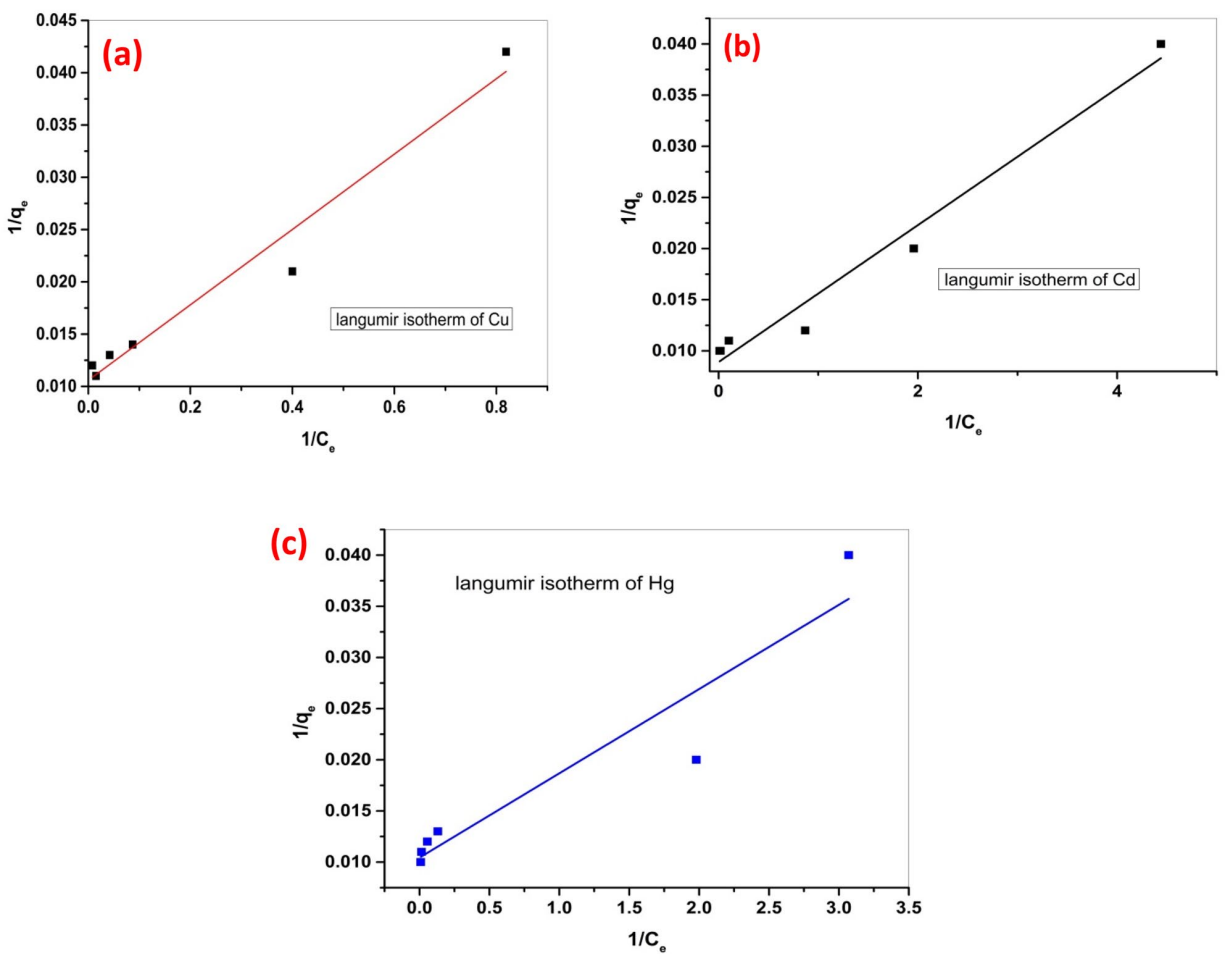
Langmuir isotherm of metal ions (**a**) Langmuir model of Cu (II) adsorption, (**b**) Langmuir model of Cd (II) and (**c**) Langmuir model of Hg (II).

The Langmuir isotherm represents an exemplary localized monolayer model wherein the solid adsorbent is characterized by a constrained quantity of precisely defined active sites that are uniformly distributed in energy. This model posits the absence of transmigration or lateral interaction among the molecules that were adsorbed, as presented in Fig. 3.

**Figure 4:**
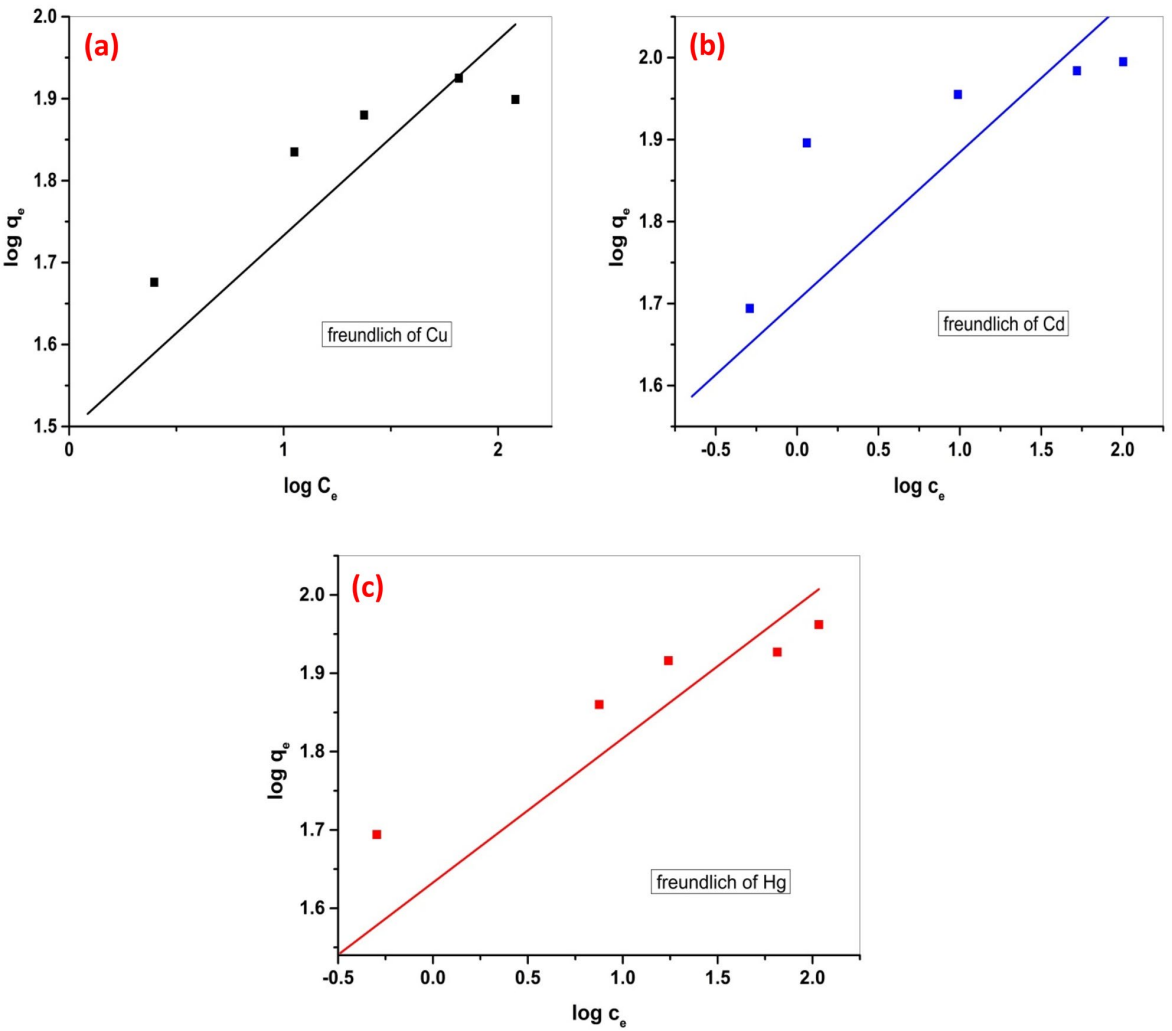
(**a**) Freundlich isotherm of Cu (II) adsorption, (**b**) Freundlich isotherm of Cd (II) adsorption and (**c**) Freundlich isotherm of Hg (II) adsorption.

Conversely, the Freundlich isotherm as shown in Fig. 4, is the most widely recognized isotherm for multiple-layer adsorption. It accounts for a non-ideal interaction involving the numerous sites that are active on the adsorbent surface, leading to subsequent binding within the linked layer and ultimately resulting in multiple layers of adsorption. The equilibrium uptake is theoretically considered to be unlimited due to the unbounded interaction at multiple layer levels.

The Langmuir isotherm model exhibits higher correlation coefficient values compared to the Freundlich isotherm model, as illustrated in (Table 1). The sorption process is well described by the Langmuir isotherm model as displayed in Fig. 3. These show that the metal ions bind to the active sites on the adsorbent surface like a monolayer. Moreover, the RL values gained for metal ions lied between 0 and 1, which indicated that the adsorption process is normal and favorable for metal ions on Cel-Gua sorbent.

**Table 1:** Isotherm parameters for the adsorption of metal ions on (Cel-Gua) sorbent.

Metal ions	Langmuir parameters				Freundlich parameters		
	q_max_ (mg/g)	K_L_ (L/mg)	R_L_	R^2^	K_F_	1/n	R^2^
Cu	94.33	0.294	0.063	0.961	31.25	0.238	0.735
Cd	112.107	1.33	0.014	0.968	50.54	0.180	0.626
Hg	95.78	1.26	0.015	0.872	42.93	0.184	0.750

### Docking studies

The molecular operating environment (MOE) software was utilized for conducting molecular docking evaluations. Access to the protein data bank was made on March 16, 2024 and the protein crystal structures (code 5OD4) were installed due to their presence in various bacterial and fungal strains. Water molecules surrounding the protein were removed, and hydrogen atoms were supplied. Subsequently, the conformation of each analyzed molecule having the lowest binding energy was improved utilizing the MMFF94x force field^39–41^. Alpha site spheres were created through MOE's site finder tool. The free binding energy (S, kcal/mol), which indicates binding affinity, was determined based on the acidic side chain interactions between the compound and protein. Through docking with the co-crystalline Cel-Gua chelate, our docking methodology was validated, yielding a root mean square deviation (RMSD) value of 2.2999 kcal/mol for the Cel-Gua compound.

## Results

### Characterization of Cel-Gua

**Figure 5:**
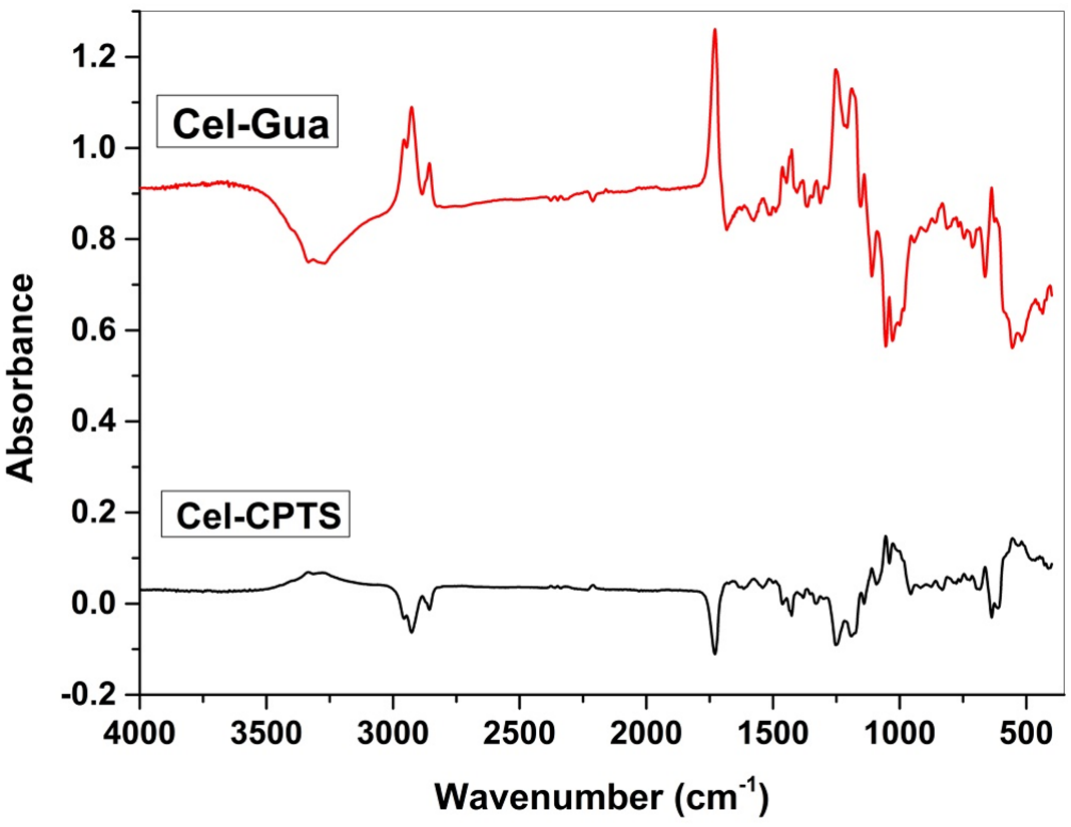
Infrared spectra of cellulose with a silylating agent (Cel-CTPS) and cellulose modified with the complexing agent, diaminoguanidine (Cel-Gua).

The Cel-Gua was distinguished by utilizing infrared spectroscopy, and the findings are depicted in Fig. 5. Like illustrated in the graphic, the interpretation of the spectrum was impeded through the overlapping of cellulose patterns with patterns corresponding to vibrations of the group molecules in the ligand. The Cel-CPTS treated with the silylating agent exhibited the appearance of the band within the vicinity of 663 cm^−1^. This particular band is ascribed to the vibrations of the CꟷCl bond in the silylating agent molecule, confirming the taking place of the reaction. Subsequent to the interaction of Cel-CPTS with aminoguanidine, the band dissipates due to the binding occurring through a nucleophilic assault of the nitrogen atom on the atom of chlorine, which is eliminated from the molecule. The mechanism of reaction demonstrated in Fig. 1 indicates that the association took place through groups of amine, forming a (C–NH–C) secondary amine bond. Consequently, an absorbing band is expected to emerge in the region of 3300 cm^−1^, confirming the completion of the reaction^42,43^.

**Figure 6:**
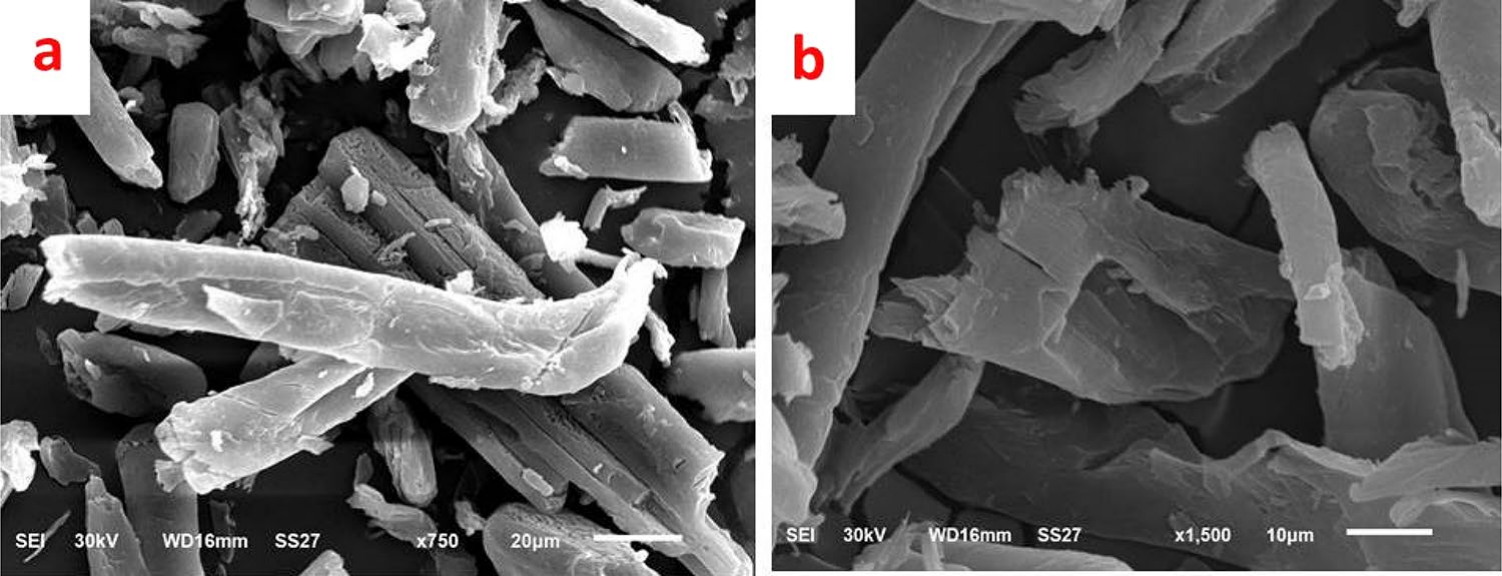
SEM images of (**a**, **b**) Cel-Gua sorbent.

The images from SEM Fig. 6 were taken to examine the surface morphology of the Cel-Gua adsorbent and give real-time observation of the sample surface's smooth and regular rod structure. The SEM image shows that after incorporating the complexing agent, the porosity of the Cel-Gua sorbent increased, as its surface area; which elevated the number of active adsorption sites for metal ion binding ^42^.

**Figure 7:**
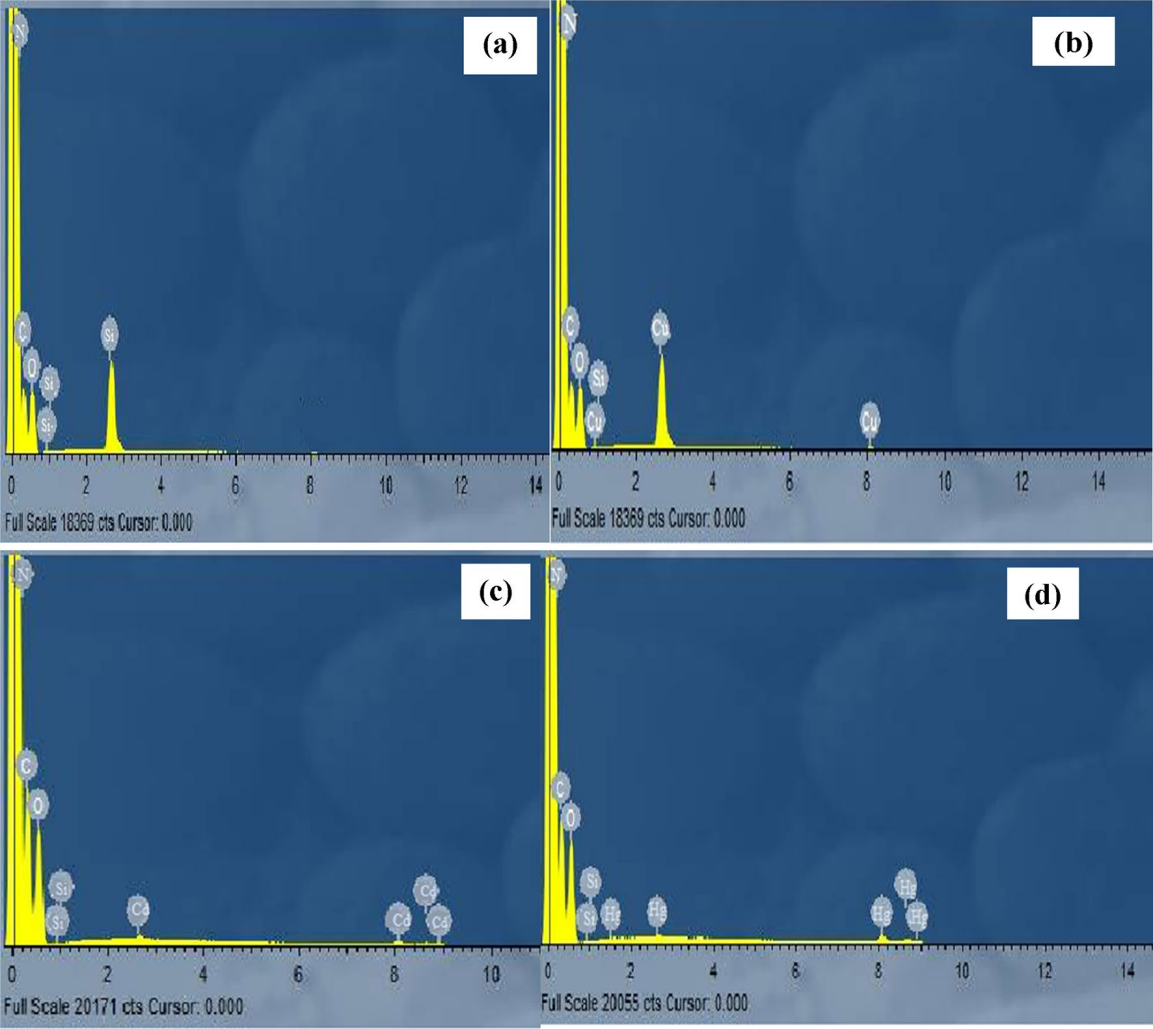
EDS images of: (**a**) Cel-Gua before adsorption, and (**b**, **c**, **d**) after adsorption of metal ions Cu (II), Cd (II), Hg (II), respectively.

Electrons dispersive X-ray spectroscopy (EDS) was used to confirm the adsorption of the metal ions on Cel-Gua fibers and the results are shown in Fig. 7. The presence of nitrogen peak confirms the presence of diaminoguanidine compound on the silanized cellulose. Furthermore, the presence of the peaks which are related to Cu (II), Cd (II) and Hg (II) ions indicates the adsorption of these metal ions on Cel-Gua chelating fibers.

**Figure 8:**
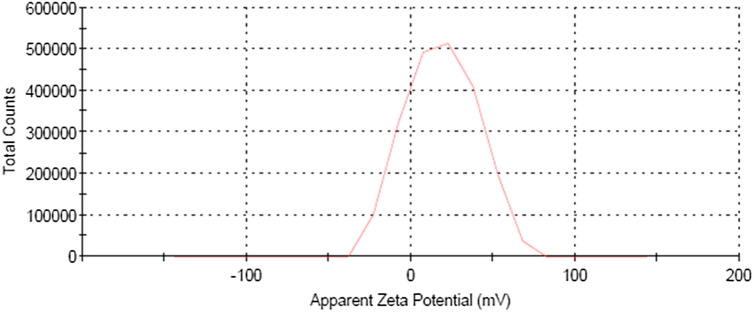
Zeta potential of Cel-Gua component.

The stability of Cel-Gua particles in the dispersion media relies on their surface charge. The presence of electrostatic repulsion forces between particles carrying identical charges prevents their aggregation. Determining the surface charge density directly poses a challenge; therefore, the measurement of zeta potential, indicating the level of repulsion among like charges, is essential to assessing particle stability in the medium. Typically, Cel-Gua particles exhibit stability in the dispersion media while the zeta potential is below − 15 mV or above + 15 mV. A higher zeta potential value corresponds to increased repulsion among Cel-Gua particles, enhancing their stability in the medium ^44^. The measured zeta potential of Cel-Gua particles was + 18.7 mV as shown in Fig. 8, confirming their stability.

**Figure 9:**
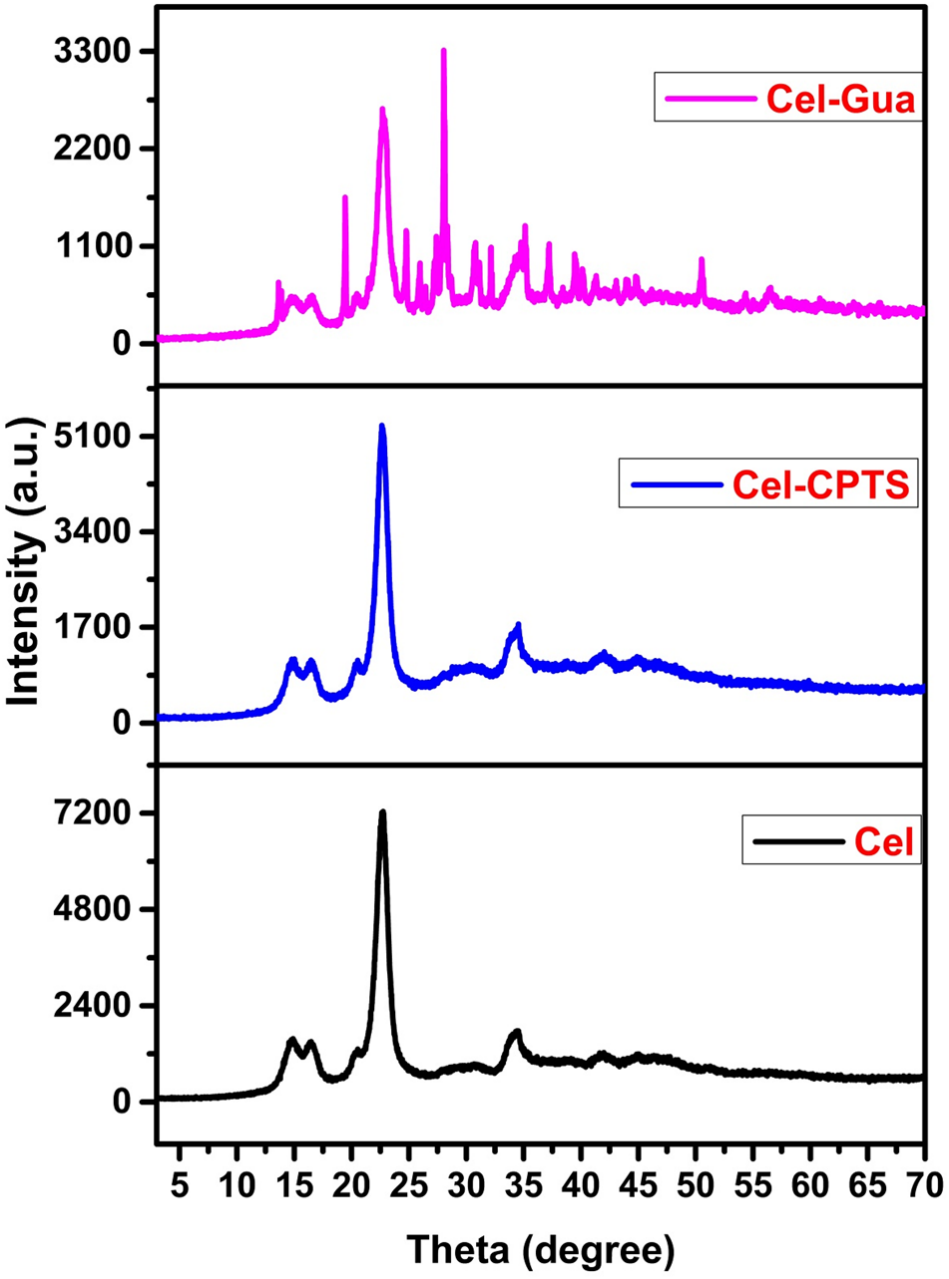
XRD pattern of Cel, Cel-CPTS and Cel-Gua structure.

XRD pattern of Cel, Cel-CPTS and Cel-Gua are shown in Fig. 9. The XRD patterns illustrate the typical peak of crystalline cellulose at 22.74° that relates to the (200) reflection. Moreover, the less intense peaks arise at about 14.61°,16.38°, and 34.60° are the characteristic of (1$\overline{1}$0), (110) and (004) reflections, respectively. The crystallinity index (CrI) of the samples was calculated according to the Segal method^45^.

$$ {\text{CrI}} = \frac{{I_{200 } - I_{am} }}{{I_{200} }} \times {1}00 $$

where I_200_ is the intensity of the crystal peak at the maximum 2θ = 22.74° and I_am_ is the intensity at the minimum 2θ = 14.61°. The crystallinity indexes of Cel, Cel-CPTS and Cel-Gua are 79.07, 79.33, and 78.62%, respectively. These results reveal that the ordered structure of crystalline cellulose is not significantly altered after the modification process of cellulose fibers^46,47^.

**Figure 10:**
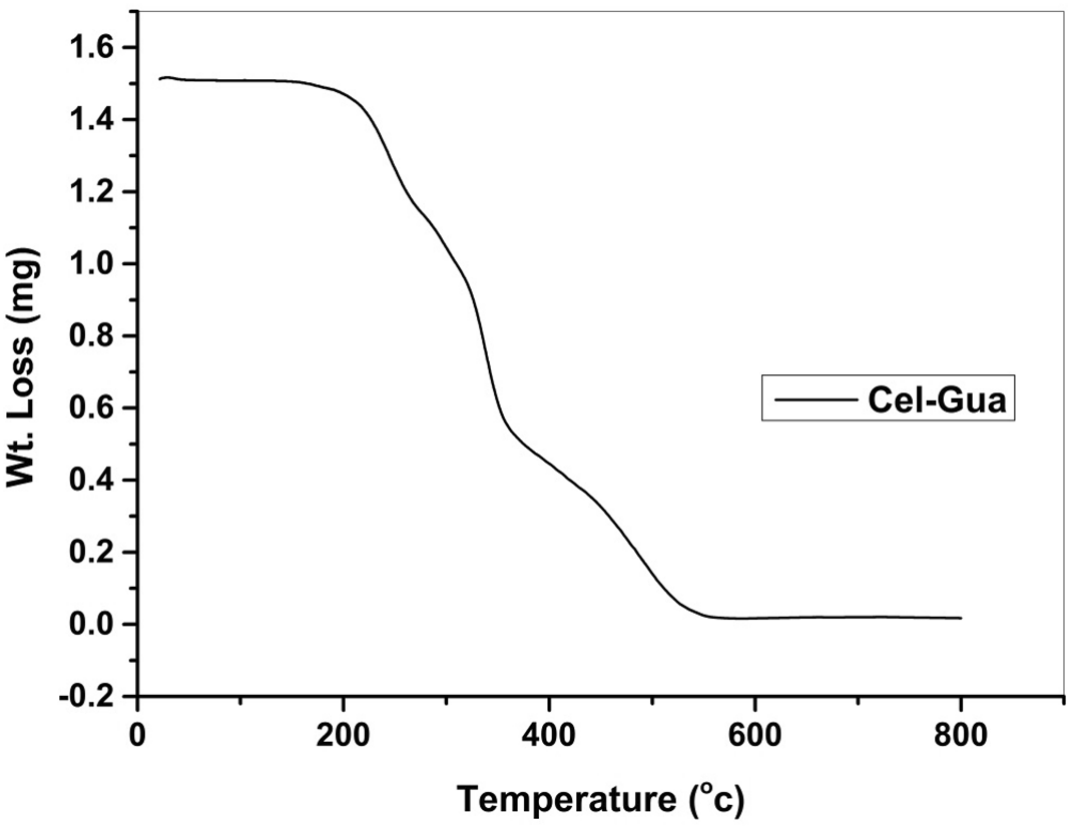
TGA curve of Cel-Gua structure.

The thermal performance of Cel-Gua powder was assessed through TGA, as illustrated in Fig. 10. During the lower temperature range, the elimination of moisture and volatile components took place, while at higher temperatures, specific thermochemical reactions occurred, leading to the degradation of the polymer material. Cel-Gua powder encountered a reduction in mass by 6% within the temperature interval of 25–217 °C as a result of the elimination of the water layer that was absorbed and the surface hydroxyl groups from the surface. Whereas in the 220–448 °C range, a 22% loss of mass was observed, which could be assigned to the decomposition and deacetylation of the cellulose backbone within the complex beads. It was noted that between 450 and 800 °C, the decomposition process slowed down, leading to a minimal 2% mass loss, indicating the presence of a robust chemical bond between Cel-Gua particles that provided stability to the complex beads even under high temperatures^48,49^.

The elemental analysis was evaluated for cellulose, Cel-CPTS, and Cel-Gua compounds to validate the modification step. The results, depicted in Table 2, indicate an increase in the nitrogen percentage from 0 to 16.4890% in the Cel-Gua sample, with a decrease in the carbon atom percentage from 42.50 to 32.706% and the hydrogen atom percentage from 6.074 to 5.857%. The diaminoguanidine modified agent, having the molecular formula CH7N5, its reaction with the silanized cellulose resulted in the insertion of nitrogen groups, which led to an increase in the nitrogen percentage in the modified component. So, these results prove the formation of the Cel-Gua material by reacting silanized cellulose with diaminoguanidine. The concentration of the inserted a diaminoguanidine units was calculated to be approximately 1.55 mmol g^−1^.

**Table 2:** Elemental analysis of Cel, Cel-CPTS and Cel-Gua.

Sample	C%	H%	N%
Cel	42.7734	5.9826	0
Cel-CPTS	42.5076	6.07422	0
Cel-Gua	32.7063	5.8572	16.4890

### Molecular docking

**Figure 11:**
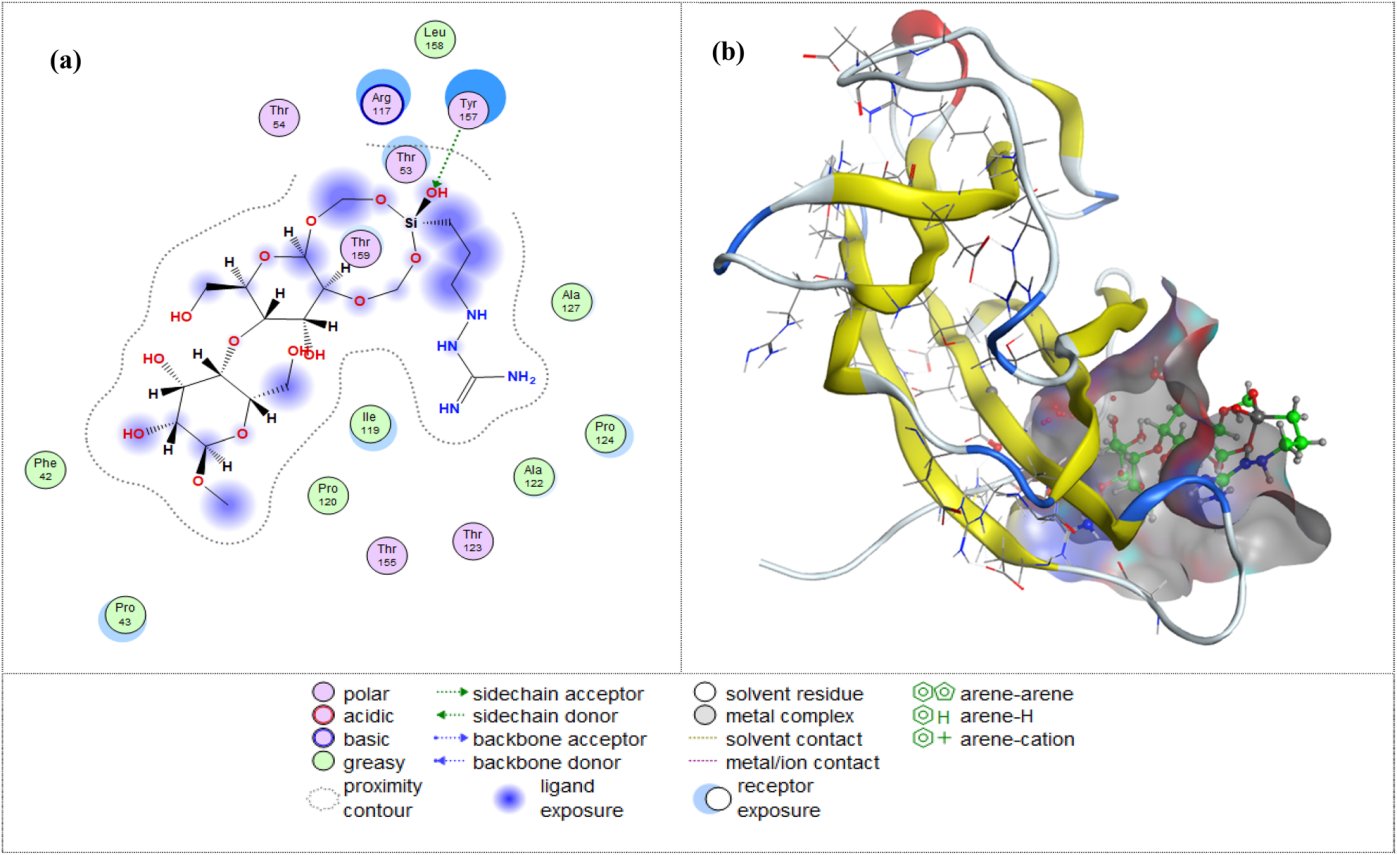
The 2D and 3D docking interaction of the Cel-Gua compound chelate with protein (protein data bank [PDB] code = 5OD4).

Molecular docking allows us to better examine the potential mechanisms of interaction and binding affinity of successful therapeutic medications, demonstrating how effectively novel chemical compounds carry out bioactivity against the target ^50^. In accordance with molecular docking and protein binding interactions with a protein (ID: 5OD4) obtained from the Protein Data Bank. The Cel-Gua (Fig. 11) forms attractive bonds with Tyr 157 as a side chain acceptor and interaction with Thr 159 as a free binding energy (S = − 6.3875 kcal/mol) and the root mean square deviation (RMSD = 2.2999 kcal/mol). This result indicates that the Cel-Gua has a higher docking score. Figure 11 shows all of the 2D and 3D interactions between the Cel-Gua chemical compound and the 5OD4 protein.

### Adsorption study on Cel-Gua

#### Effect of pH

**Figure 12:**
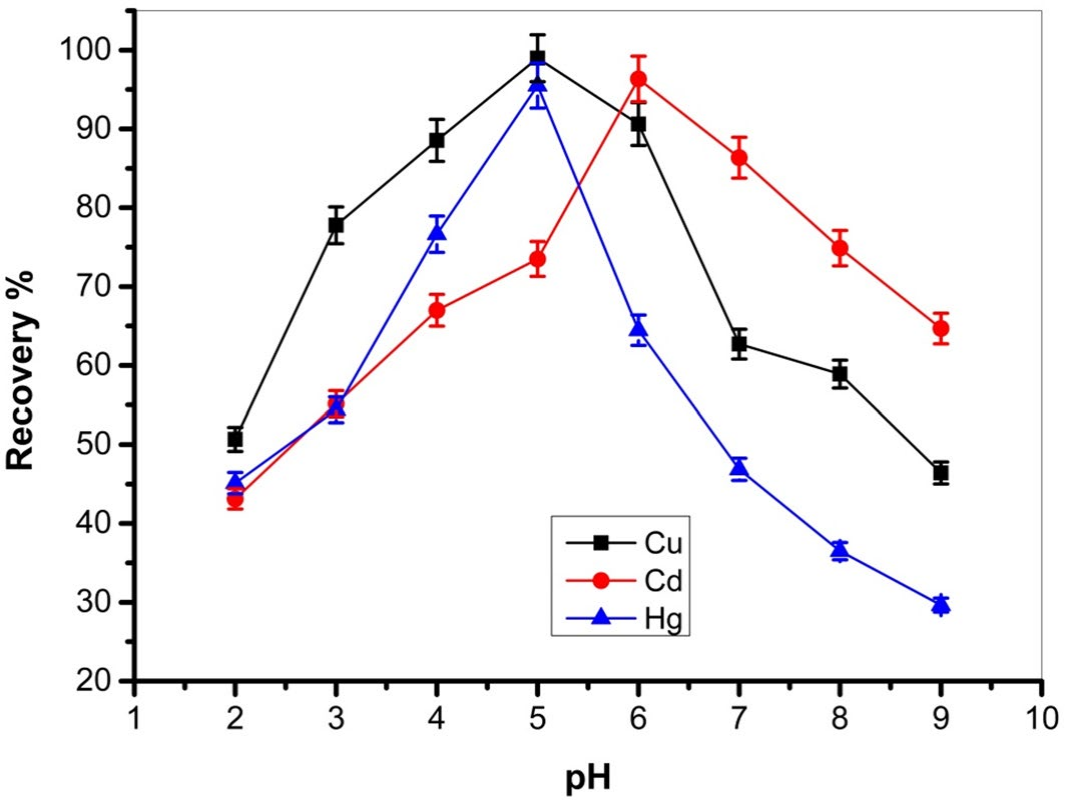
Effect of solution pH for the adsorptive uptake of Cu (II), Cd (II) and Hg (II) onto Cel-Gua complex.

The pH influences all of the surface charge on Cel-Gua and the metal ions in the solution; thus, it has been established as a significant variable in the process of metal removal. The pH influence on the adsorption of Cu (II), Cd (II), and Hg (II) onto the Cel-Gua was investigated within the range of 2–9, as depicted in Fig. 12. The significant influence of pH was particularly noticeable on the metal ion adsorption at pH 4–7, while at pH > 7, the adsorption of metal ions began to decrease. This elimination in adsorption at pH > 7 would be related to electrostatic repulsion among Cel-Gua and metal ions. So the maximum adsorption was noted at pH 5 for Cu (II) and Hg (II) ions, but Cd (II) ions have maximum adsorption at pH 6.

#### Effect of initial concentration

**Figure 13:**
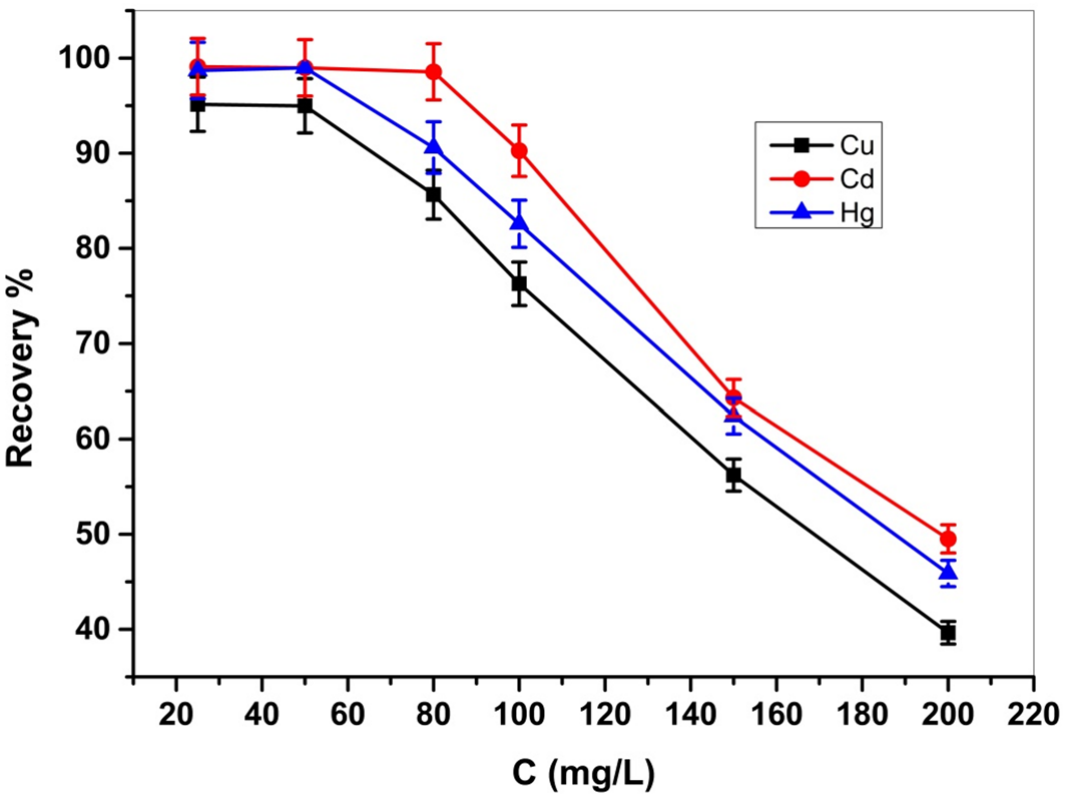
Effect of initial concentration of Cu (II), Cd (II) and Hg (II) on adsorption process.

The influence of the initial concentrations of metal ions on the adsorption process of the investigated analytes, was examined in the range of (25–200 ppm), as presented in Fig. 13, utilizing a 0.05 g Cel-Gua adsorbent and a suitable pH value for each metal ion while maintaining all other parameters unchanging, shaking for 4 h at room temperature. The graph shows that the adsorption recovery of Cel-Gua rises with the increasing initial concentration of metals at 50 ppm for Cu (II), Hg (II), and 80 ppm for Cd (II) ions, but decreases at higher concentrations. Adsorption efficiency increased with initial metal concentrations because of the presence of a large number of active binding sites on the surface of Cel-Gua. At higher concentrations of metal ions, the adsorption efficiency approached its maximum value and then decreased indicating that the active binding sites on the Cel-Gua surface were approaching to saturation.

#### Effect of dose

**Figure 14:**
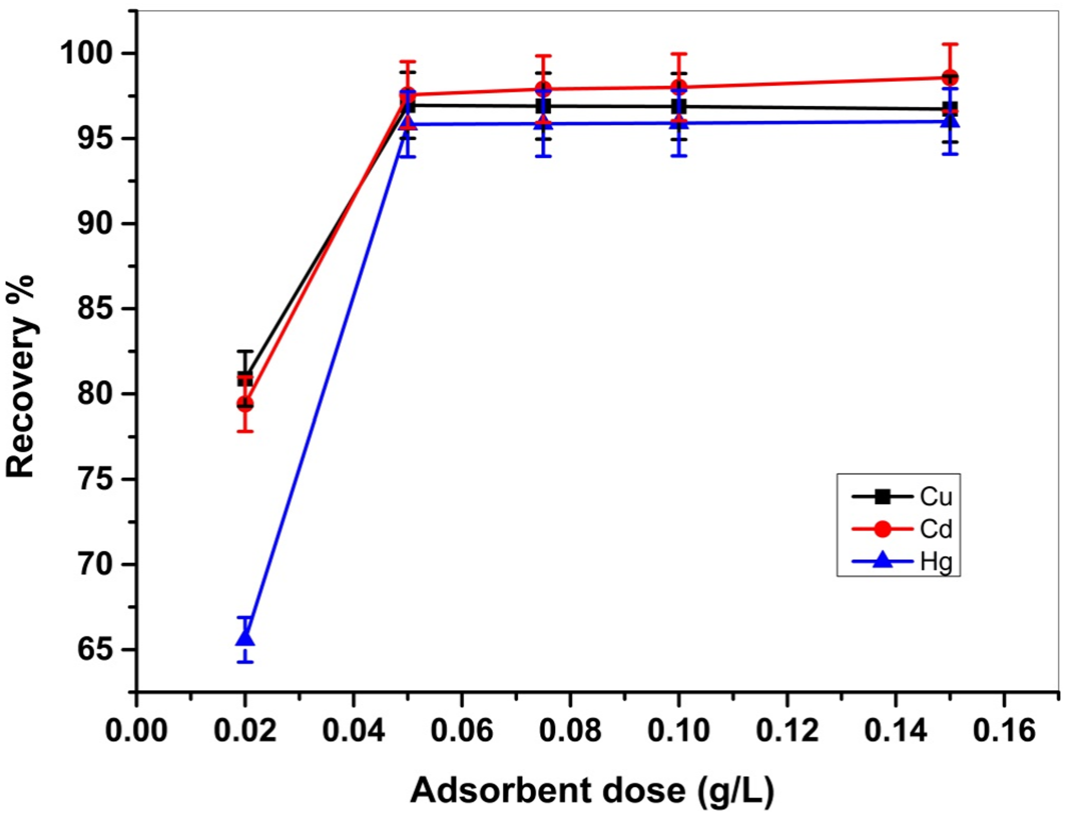
Effect of dose on Cu (II) (50 ppm), Cd (II) (80 ppm) and Hg (II) (50 ppm), adsorption on Cel-Gua at room temperature for 4 h.

The impact of the dose of Cel-Gua (Fig. 14) was evaluated within the range of (0.02–0.15 g) while keeping the other parameters constant, shaking for 4 h at room temperature, and employing a 50 ml of 50 ppm for Cu (II), Hg (II), and 80 ppm for Cd(II) at an adequate pH value for each metal ion. The best adsorption efficiency for all metals was achieved with 0.05 g of Cel-Gua in 50 ml of metal solutions.

#### Effect of adsorption time and adsorption kinetics

**Figure 15:**
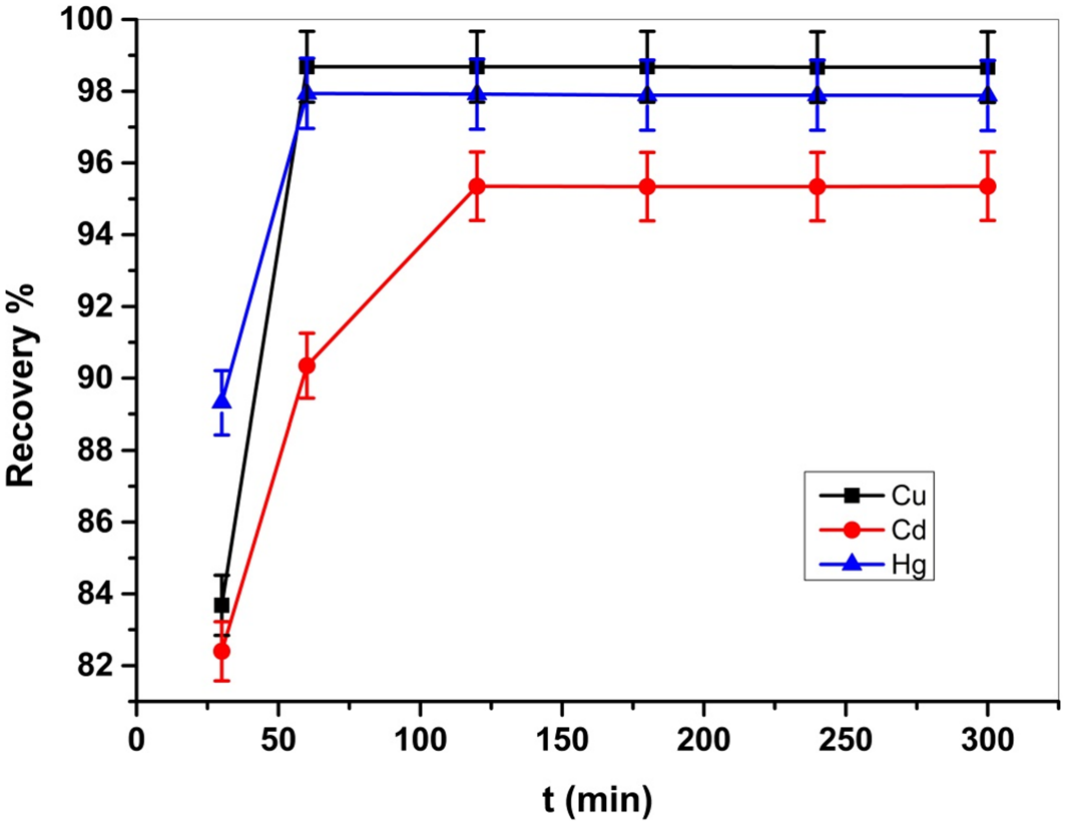
Effect of time on Cu (II), Cd (II) and Hg (II) adsorption on Cel-Gua sorbent.

To investigate the impact of reaction time on the metal adsorption process, the time was adjusted into the range of 30–300 min employing 0.05 g Cel-Gua and an adequate amount of concentration for each metal ion 50 ppm of Cu (II), Hg (II), and 80 ppm of Cd (II) at a suitable pH value for each metal using 50 ml of metal solutions at room temperature. Figure 15 revealed that the adsorption efficiency was fast around 80% in the first 30 min, then by raising the period from 30 to 60 min, the adsorption efficiency jumped to 98% for Cu (II) and Hg (II) ions and remained constant, but for Cd (II) ion, it increased to 95% after rising time to 120 min, and then the adsorption efficiency kept constant, which indicates that it achieved equilibrium^51^.

**Figure 16:**
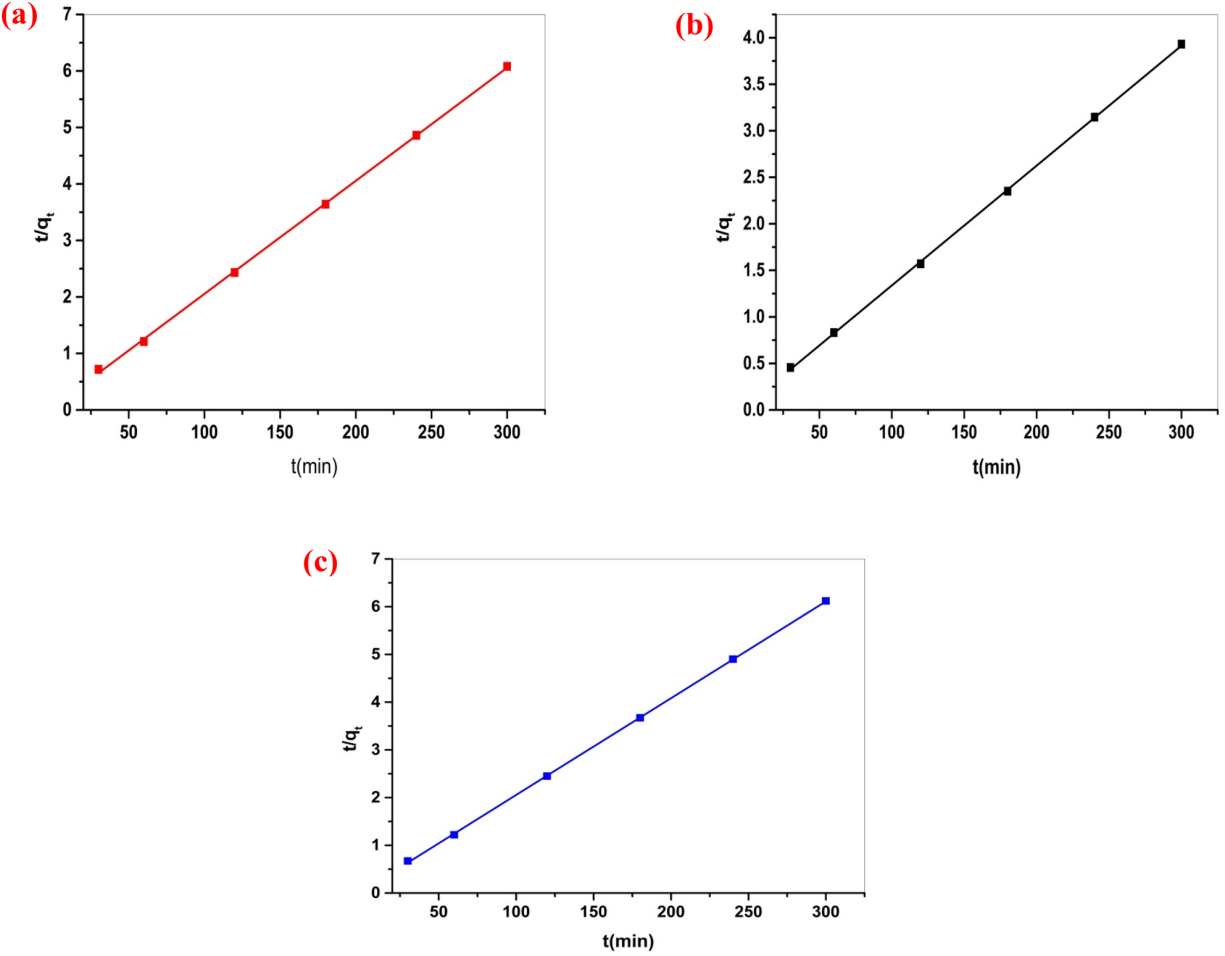
(**a**) Pseudo 2nd order for Cu (II) adsorption, (b) Pseudo 2nd order for Cd (II) adsorption, and (**c**) Pseudo 2nd order for Hg (II) adsorption.

**Figure 17:**
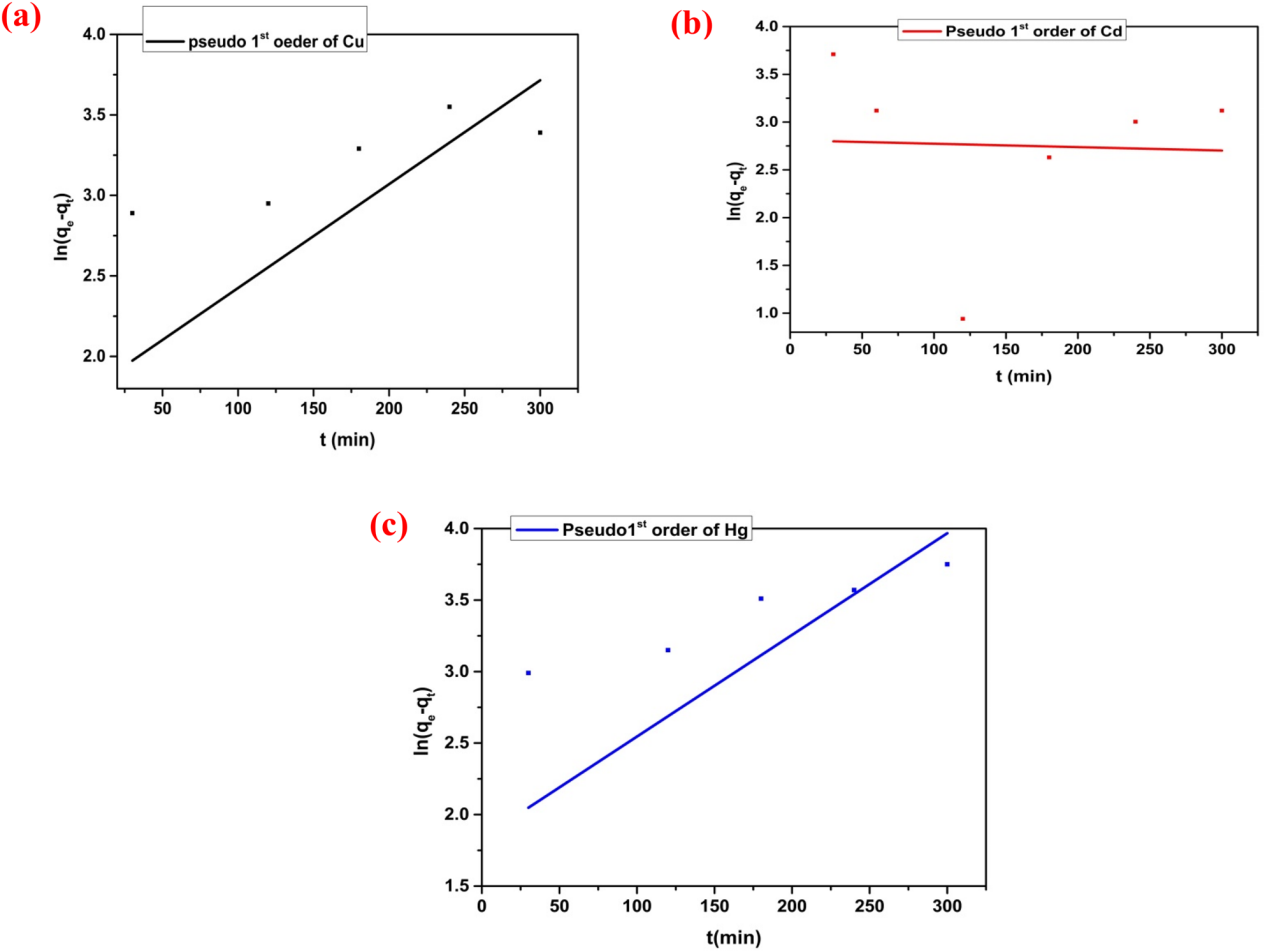
(**a**) Pseudo 1st order for Cu (II) adsorption, (**b**) Pseudo 1st order for Cd (II) adsorption, and (**c**) Pseudo 1st order for Hg (II) adsorption.

Adsorption kinetic studies could determine the mechanism of the adsorption process as well as the adsorbent's physical or chemical characteristics ^52^. Consequently, to explore the adsorption of Cu (II), Cd (II), and Hg (II) ions. Actually, the adsorption of heavy metal ions onto porous materials takes place via three steps; (i) diffusion of the metal ions from the bulk of solution to the adsorbent surface; (ii) diffusion of the metal ions into the material pores; (iii) chemical adsorption of the metal ions on the active sites of the adsorbent material. The validity of the pseudo-second-order equation may support the surface chemical adsorption as a rate-controlling mechanism. Two models were established: pseudo-second-order and pseudo-first-order. The PSO kinetic model was explained in its linear form, as illustrated in Fig. 16, but the first-order kinetic model was explained in its nonlinear form, as explained in Fig. 17. The obtained parameters for every model are presented in Table 3. Both of the kinetic models usually demonstrate a surface-controlled adsorption mechanism. In this investigation, the PSO model fits more accurately than the PFO model, with a correlation coefficient R^2^ equal to 0.999, as illustrated in Table 3. Therefore, the chemisorption process controls the adsorption of Cu (II), Cd (II), and Hg (II) ions on Cel-Gua surface.

**Table 3:** Kinetic parameters for the adsorption of Cu (II), Cd (II) and Hg (II) onto Cel-Gua sorbent.

Metal ions	First-order model				Second-order model		
	q_e exp._ (mg/g)	q_e_,_cal._ (mg/g)	K_1_ (min^−1^)	R^2^	K_2_	q_e_,_cal_ (mg/g)	R^2^
Cu (II)	49.34	5.92	1.07 × 10^–4^	0.226	0.007	49.95	0.999
Cd (II)	76.28	16.59	− 0.029	− 0.248	0.003	78.12	0.999
Hg (II)	48.97	6.258	1.18 × 10^–4^	0.268	0.014	49.30	0.999

#### Effect of temperature and thermodynamic studies

**Figure 18:**
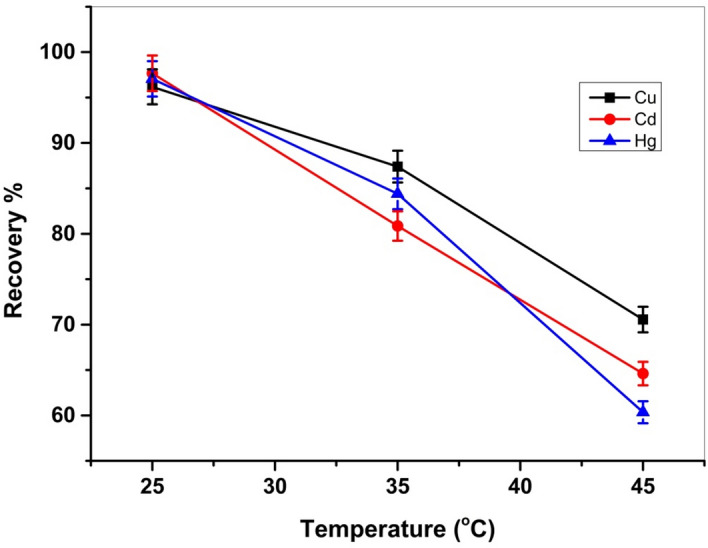
Effect of temperature on Cu (II), Cd (II) and Hg (II) adsorption on to Cel-Gua adsorbent.

To investigate the impact of temperature on Cel-Gua adsorption efficiency, isotherms were obtained within the range of 298 to 318 K. Equilibrium isotherms at the examined temperatures are displayed in Fig. 18. It was noted from Fig. 18 that the adsorption efficiency of Cel-Gua reduced with rising temperature.

To investigate the adsorption of metal ions onto the Cel-Gua sorbent surface in regards to spontaneity and possibility and to establish the degree of randomization during solid/liquid extraction, adsorption thermodynamic factors were established. The adsorption of metal ions was examined at various temperatures, from room temperature to 45 °C, at pH 5 for Cu (II) and Hg (II) for 60 min, and Cd (II) at pH 6 for 120 min. The free energy variables (ΔG°_ads_), adsorption entropy factor (ΔS°_ads_), and heat of enthalpy variable (ΔH°_ads_) for metal ion adsorption by Cel-Gua adsorbent were measured. The ΔG°_ads_ factor was determined using the following Eqs. (5), (6), and (7).

5 $$ {\text{K}}_{{\text{L}}} = {\text{q}}_{{\text{e}}} /{\text{C}}_{{\text{e}}} $$

6 $$ \Delta {\text{G}}^{{\text{o}}} = - {\text{RTlnK}}_{{\text{L}}} $$

7 $$ \Delta {\text{G}}^{{\text{o}}} = \Delta {\text{H}}^{{\text{o}}} - {\text{T}}\Delta {\text{S}}^{{\text{o}}} $$

**Figure 19:**
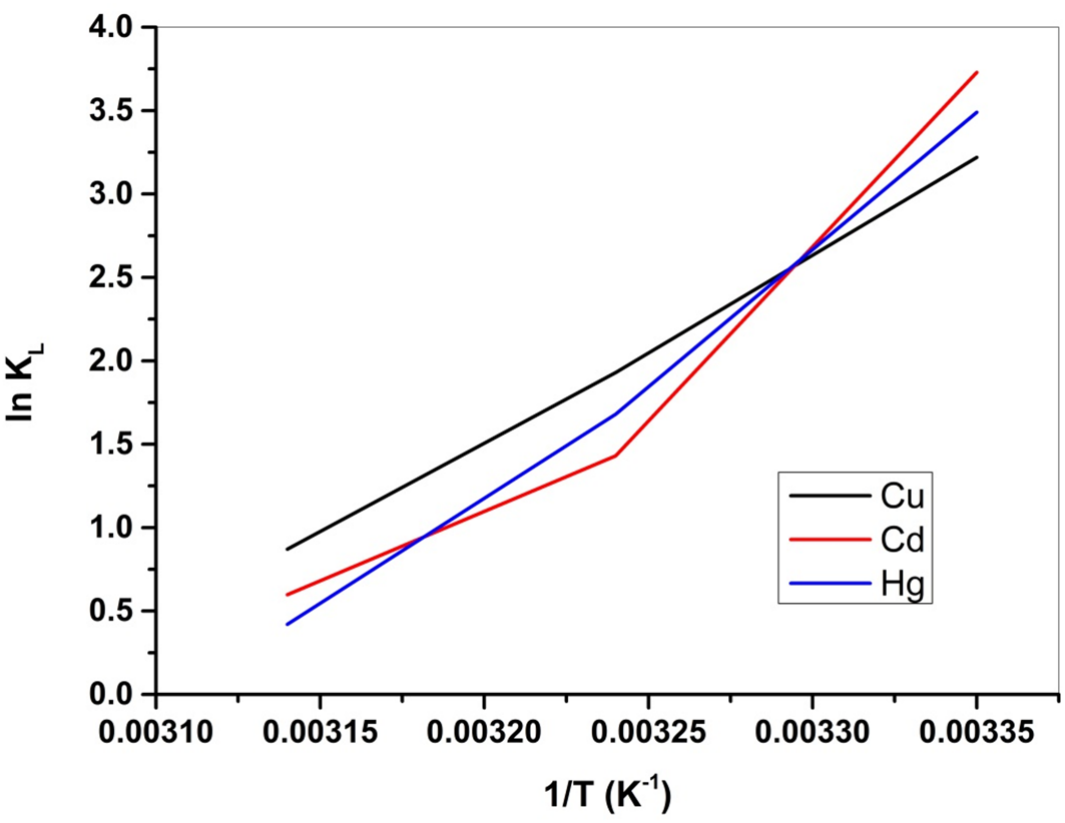
Plot of ln K_L_ versus (1/T) absolute temperature for the adsorption of Cu (II), Cd (II) and Hg (II) on to Cel-Gua adsorbent.

K_L_ represents the thermodynamic constant at equilibrium, where q_e_ signifies the concentration of metal ions adsorbed by Cel-Gua material at equilibrium (mg/g), C_e_ denotes the metal ion equilibrium concentration (mg/L), and R stands for the universal gas constant (8.314 J mol^−1^ K^−1)^. The remaining parameters (ΔH° and ΔS°) were derived from the plot of 1/T vs. ln K_L_, where the slope relates to (− ΔH°/R) and the intercept relates to (ΔS°/R), as illustrated in Fig. 19. Analysis of the experimental data presented in Table 4 revealed a negative value for ΔG°, indicating that the adsorption of metal ions by the Cel-Gua complex occurs spontaneously. Furthermore, the negative value ΔH° suggests that the metal ion adsorption by the Cel-Gua sorbent is exothermic. The negative values ΔS° indicate that the metal ion adsorption onto the Cel-Gua surface results in decreased disorder and better arrangement^53,54^. The values of (ΔH^o^_ads_) < 8 kJ/mol is representative of physical sorption and (ΔH^o^_ads_) > 8–16 kJ/mol is due to chemical sorption^55^. For adsorption of studied metals on Cel-Gua, the values of (ΔH^o^_ads_) are found above 8, so adsorption of studied metals on Cel-Gua occurred on the surface by chemical bonding between sorbent and sorbate.

**Table 4:** Thermodynamic parameters for the adsorptive uptakes of Cu (II), Cd (II) and Hg (II) onto Cel-Gua sorbent.

Metal ions	Temperature (K)	∆G (kJ/mol)	∆H (kJ/mol)	∆S (J/mol.K)
Cu (II)	298	− 7.97	− 93.07	− 285.17
	308	− 4.94		
	318	− 2.30		
Cd (II)	298	− 9.24	− 124.82	− 388.84
	308	− 3.66		
	318	− 1.58		
Hg (II)	298	− 8.64	− 121.79	− 379.53
	308	− 4.30		
	318	− 1.110		

### Reusability and regeneration studies

**Figure 20:**
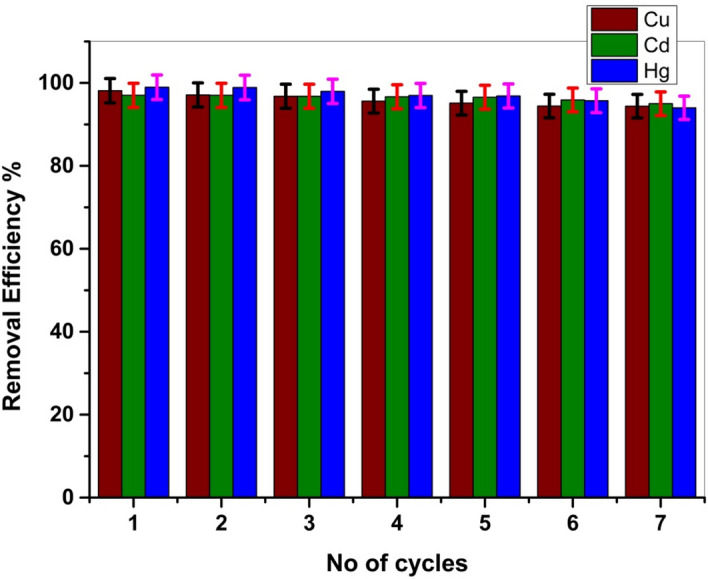
Regeneration efficiency of Cel-Gua adsorbent.

Reusability can be performed by different eluents for the desorption of metal ions from the Cel-Gua biosorbent, like ethanol, HCl (0.2 M), NaHCO_3_ (0.1 M), NaOH (0.2 M), HNO_3_ (0.2 M), and K_2_CO_3_ (0.1 M). It was found that HNO_3_ (0.1 M) was the most effective and successfully used for elution of the studied metal ions (adsorbates) from Cel-Gua biosorbent at room temperature. The reusability of Cel-Gua biosorbent was tested for seven cycles of sorption–desorption phases at the best conditions. Observation from Fig. 20 after seven cycles, Cel-Gua has high adsorption efficiency (more than 94%). Thus, it is foreseeable that Cel-Guah has high stability and could be a good sorbent for pre-concentration and elimination of Cu (II), Cd (II), and Hg (II) from aquatic solutions.

### Accuracy and application in alum sample

The proposed Cel-Gua sorbent was utilized for the separation and removal of Cu (II), Cd (II), and Hg (II) ions from alum sample at the best conditions. Numerous quantities of metal ions were also spiked into the alum sample. The metal ion concentrations following the adsorption process were measured using flame atomic absorption spectroscopy (FAAS). The results presented in Table 5 illustrate the accuracy of Cel-Gua sorbent for the recovery of metal ions, with recoveries ranging from 96 to 100%. Moreover, there is high agreement among the added concentration of metal ions and measured evidence that the capability of Cel-Gua sorbent for the effective recovery and measurement of Cu (II), Cd (II), and Hg (II) ions in an actual alum sample with great precision and accuracy.

**Table 5:** Analysis of Cu (II), Cd (II) and Hg (II) ions in alum sample after their separation and recovery by Cel-Gua sorbent.

Metal ions	Added (mg/L)	Measured (mg/L)	Recovery %	RSD %
Cu (II)	0.00	0.01	99.9	0.46
	0.40	0.20	98.07	0.05
	2.0	0.39	96.75	0.025
Cd (II)	0.00	0.005	99.89	0.2
	0.3	0.140	97.2	0.007
	1.0	0.19	96.66	0.05
Hg (II)	0.00	0.00	100	0.00
	0.14	0.005	98.21	0.2
	0.3	0.010	97.72	0.46

### Comparison of the proposed adsorbent (Cel-Gua) with other cited adsorbents

A comparison between the performance of the present adsorbent and other adsorbents previously reported in the literature is presented in Table 6. When comparing different adsorbents for the separation, the adsorption capacity, adsorption dose, and the adsorbent type should be taken into consideration. As shown in Table 6, the present adsorbent Cel-Gua has relatively high capacities for the recovery of Cu (II), Cd (II) and Hg (II) ions compared to other adsorbents in the literature.

**Table 6:** Comparison of adsorption capacity of Cu (II), Cd (II), and Hg (II) onto Cel-Gua with previously reported studies.

Ions	Adsorbent	Adsorbent dose	Adsorption capacity (mg/g)	References
Cd(II)	Cellulose derived from corn stalk	0.5 g	21.37	^56^
Cd(II)	Pretreated silica gel	300 mg	45.5	^57^
Hg(II)	Magnetic nanoparticles doped with 1,5-diphenylcarbazide	0.1 g	44	^58^
Hg(II)	Silica gel modified with 2-(2-oxoethyl)hydrazine carbothioamide	30 mg	37.5	^59^
Cu(II)	Cotton-DTPA	0.1 g	69.2	^60^
Cu(II)	Cationic wheat straw	0.1 g	33.5	^61^
Cu(II)	P- tert(dimethylamino)methyl-calix[4]arene	30 mg	34.4	^62^
Cd(II),Cu(II)	Azolla filiculoides (aquatic fern)	200 mg	62, 86	^63^
Cu(II)	Palm oil fruit shells	0.5 g	28–60	^64^
Cu(II), Cd(II)	Mercerized cellulose (Cell 2, Cell4)	50 mg	(56.8, 69.4), (68, 87)	^65^
Cu(II), Cd(II)	Succinylated twice-mercerized sugarcane bagasse	0.05 g	59.5, 69.4	^66^
Cu(II)	Groun nut shells	1 g	7.60	^67^
Cu(II), Cd(II), Hg(II)	Guanyl modified cellulose	0.05 g	83, 68,48	^37^
Cu(II), Cd(II), Hg(II)	Cel-Gua	0.05 g	94.33, 112.10, 95.78	Present study

## Conclusions

A novel chelating polymer was synthesized by chemically modifying silanized cellulose with diaminoguanidine. The sorbent showed notable selectivity and great adsorption capability for Cu (II), Hg (II), and Cd (II) ions at pH 5 and 6, respectively. The kinetic investigations showed that the sorption process corresponded well to the PSO model, indicating that the sorption process was achieved by chemisorption. This process is exothermic and spontaneous at various temperatures, as specified by the thermodynamic parameters. Furthermore, the isotherm data showed that the adsorption isotherms obey the equation of the Langmuir isotherm, with maximum adsorption capacities of 94.33, 112.10, and 95.78 mg/g for Cu (II), Cd (II), and Hg (II) ions, respectively. The optimum conditions are a 0.05 g dose of Cel-Gua at room temperature with a suitable pH and initial concentration for each metal ion. Furthermore, the docking behavior of the Cel-Gua compound had been studied. From molecular docking interaction, Cel-Gua forms attractive bonds with Tyr 157 as a side chain acceptor and interaction with Thr 159 as a free binding energy (S = − 6.3875 kcal/mol) and the RMSD equals (2.2999 kcal/mol). The application and reusability investigations indicated that the sorbent can be used repeatedly for at least 7 cycles with undiminished adsorption capacity and indicated that Cel-Gua sorbent has a great potential for the recovery of metal ions from various resources.

## Data Availability

All data generated or analyzed during this study are included in this published article.
